# Using rapid online survey to assess public perceptions of Covid-19 in Gambia

**DOI:** 10.11604/pamj.supp.2020.35.2.22794

**Published:** 2020-05-12

**Authors:** Mat Lowe

**Affiliations:** 1 Society for the Study of Women´s Health (SSWH), Old Yundum, Gambia

**Keywords:** Covid-19, Gambia, coronavirus, perceptions, cases

## To the editors of the Pan African Medical Journal

**Figure 1:**
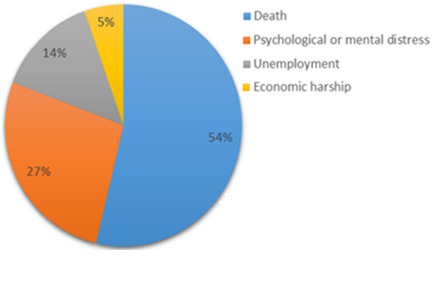
Fear towards the Covid-19 pandemic

Coronavirus disease (Covid-19), which started in Wuhan, China, in December 2019 [1,2], is an ongoing global pandemic that has led to confusion, anxiety and fear throughout the whole world [3,4]. In the Gambia, since the first case of Covid-19 was confirmed on March 17, 2020 [5], series of public health measures including case detection, contact tracing and quarantine, guidance and information to the public have been implemented. Other response strategies such as closure of schools, land borders and airspaces, travel restriction for public officials, and suspension of public gatherings have also been adopted [6-8]. However, to ensure adherence to these measures and to facilitate the prevention and management of Covid-19 in the Gambia, there is an urgent need to understand people´s perceptions of the disease, including it severity. This letter to the editors is a report of a rapid online cross-sectional survey that was conducted to assess the perceived severity of Covid-19 among adult population in the Gambia. The survey was conducted from April 4-8, 2020. It was administered through Instant Message via WhatsApp Application and using Google Doc Form. A total of 206 respondents participated in the survey. Of these, (56%) were males and (43.5%) were females. Their ages ranged from 20 to 64 years old and more than half (69%) have university education. Drawing from the data presented in (Table 1), (62.6%) of respondents reported being very worried for themselves and their family members of contracting coronavirus. (68.5%) were also very worried that there will be many cases of coronavirus in the Gambia and (70%) were not too confident that the healthcare system will be able to handle many cases of coronavirus. Most respondents (98%) believed that Covid-19 is a disease that could be spread very easily and (54%) regarded death as their biggest fear towards the Covid-19 pandemic (Figure 1).

**Table 1:** Worry about coronavirus disease (Covid-19)

Question	Response (%)
**How worried are you that you or someone in your family will be exposed to the coronavirus**	
Very worried	127 (62.6)
Somewhat worried	45 (22.2)
Not too worried	23 (11.3)
Not worried at all	8 (3.9)
**How worried are you that there will be more cases of coronavirus disease in the Gambia**	
Very worried	139 (68.5)
Somewhat worried	39 (19.2)
Not too worried	20 (9.9)
Not worried at all	5 (2.5)
**How confident are you that the healthcare system in the Gambia will be able to handle many cases of coronavirus disease**	
Not too confident	143 (70.4)
Somehow confident	51 (25.1)
Not too confident	9 (4.4)
**If a person is sick with coronavirus, how easily is coronavirus disease spreads from that person to another**	
Disease spreads very easily	199 (98%)
Disease spreads not very easily	4 (2%)

Note: % represents respondents who provided responses

## Conclusion

Although the findings reported in this letter to the editors were self-reported and limited to individuals with a higher level of education, the findings showed high level of worry and fear related to Covid-19 among adult Gambian population. This reality must be considered when communicating risks and providing guidance and information to the public, as revealed by a previous study that with high level of fear, individuals may not think clearly and rationally when reacting to Covid-19 [9]. The findings also revealed that a significant number of respondents (70%) had less confidence in the Gambian healthcare system capacity to handle many cases of coronavirus. Ensuring confidence in the healthcare system was a challenge during the Ebola virus disease response and recovery efforts [10], which must also be taken into consideration in current Covid-19 response strategies and interventions in the Gambia.

## Competing interests

The author declares no competing interests.
